# Two-Photon Na^+^ Imaging Reports Somatically Evoked Action Potentials in Rat Olfactory Bulb Mitral and Granule Cell Neurites

**DOI:** 10.3389/fncel.2017.00050

**Published:** 2017-02-28

**Authors:** Tiffany Ona-Jodar, Niklas J. Gerkau, S. Sara Aghvami, Christine R. Rose, Veronica Egger

**Affiliations:** ^1^Neurophysiology, Institute of Zoology, Universität RegensburgRegensburg, Germany; ^2^Institute of Neurobiology, Heinrich-Heine-Universität DüsseldorfDüsseldorf, Germany; ^3^School of Electrical and Computer Engineering, University of TehranTehran, Iran; ^4^School of Cognitive Science, Institute for Research in Fundamental ScienceTehran, Iran; ^5^Regensburg Center of Neuroscience, Universität RegensburgRegensburg, Germany

**Keywords:** olfactory bulb, granule cell, mitral cell axon, active dendrites, sodium transient, two-photon imaging, SBFI

## Abstract

Dendrodendritic synaptic interactions are a hallmark of neuronal processing in the vertebrate olfactory bulb. Many classes of olfactory bulb neurons including the principal mitral cells (MCs) and the axonless granule cells (GCs) dispose of highly efficient propagation of action potentials (AP) within their dendrites, from where they can release transmitter onto each other. So far, backpropagation in GC dendrites has been investigated indirectly via Ca^2+^ imaging. Here, we used two-photon Na^+^ imaging to directly report opening of voltage-gated sodium channels due to AP propagation in both cell types. To this end, neurons in acute slices from juvenile rat bulbs were filled with 1 mM SBFI via whole-cell patch-clamp. Calibration of SBFI signals revealed that a change in fluorescence Δ*F*/*F* by 10% corresponded to a Δ[Na^+^]_i_ of ∼22 mM. We then imaged proximal axon segments of MCs during somatically evoked APs (sAP). While single sAPs were detectable in ∼50% of axons, trains of 20 sAPs at 50 Hz always resulted in substantial Δ*F*/*F* of ∼15% (∼33 mM Δ[Na^+^]_i_). Δ*F*/*F* was significantly larger for 80 Hz vs. 50 Hz trains, and decayed with half-durations τ_1/2_ ∼0.6 s for both frequencies. In MC lateral dendrites, AP trains yielded small Δ*F*/*F* of ∼3% (∼7 mM Δ[Na^+^]_i_). In GC apical dendrites and adjacent spines, single sAPs were not detectable. Trains resulted in an average dendritic Δ*F*/*F* of 7% (16 mM Δ[Na^+^]_i_) with τ_1/2_ ∼1 s, similar for 50 and 80 Hz. Na^+^ transients were indistinguishable between large GC spines and their adjacent dendrites. Cell-wise analysis revealed two classes of GCs with the first showing a decrease in Δ*F*/*F* along the dendrite with distance from the soma and the second an increase. These classes clustered with morphological parameters. Simulations of Δ[Na^+^]_i_ replicated these behaviors via negative and positive gradients in Na^+^ current density, assuming faithful AP backpropagation. Such specializations of dendritic excitability might confer specific temporal processing capabilities to bulbar principal cell-GC subnetworks. In conclusion, we show that Na^+^ imaging provides a valuable tool for characterizing AP invasion of MC axons and GC dendrites and spines.

## Introduction

Many neuronal interactions in the vertebrate olfactory bulb are mediated by dendrodendritic synapses at both stages of the olfactory network, the glomerular input layer and the EPL. Accordingly, the dendrites of many classes of olfactory bulb neurons dispose of several mechanisms that support dendrodendritic processing, including release of transmitter (Sheperd et al., 1990; Wachowiak and Shipley, 2006). In particular, bulbar dendrites often are endowed with high densities of active conductances since they feature an efficient propagation of action potentials.

The principal MCs and the axonless GCs are especially interesting because they interact via dendrodendritic reciprocal synapses and thus both rely on dendritic transmitter release to exchange information. The specific functional consequences of this local interaction are not fully resolved yet. MC dendrites are accessible to whole-cell patch-clamp; in combination with Ca^2+^ and voltage-sensitive dye imaging it has been shown that (1) action potentials can be initiated in their apical dendritic tuft and then propagate to the soma and that (2) under certain conditions action potentials will travel far out into their lateral dendrites (Chen et al., 1997; Margrie et al., 2001; Xiong and Chen, 2002; Christie and Westbrook, 2003; Debarbieux et al., 2003; Djurisic et al., 2004).

So far, propagation in GC dendrites has been investigated only indirectly via Ca^2+^ imaging (Egger et al., 2003; Zelles et al., 2006), since the small sizes of their dendrites and soma have precluded dendritic patching and voltage-sensitive dye imaging. Therefore other techniques such as Na^+^ imaging might yield useful additional information on their active properties. This approach has been introduced by Jaffe et al. (1992) and Ross et al. (1993), who used the fluorescent Na^+^-indicator SBFI for detection of Na^+^ influx through voltage-gated sodium channels (Na_v_) and thereby provided first direct evidence for active backpropagation of action potentials into dendrites of hippocampal CA1 pyramidal neurons. Later on, two-photon imaging with SBFI demonstrated the existence of Na^+^ transients in response to backpropagating action potentials in dendritic spines of CA1 neurons (Rose et al., 1999). Na^+^ imaging also allows detection of Na^+^ influx into active axons (Kole et al., 2008; Bender and Trussell, 2009; Fleidervish et al., 2010; Scott et al., 2014; Miyazaki and Ross, 2015).

Olfactory bulb GCs represent a special case since they do not bear a classical axon. Rather, their only output occurs onto MC and tufted cell (TC) lateral dendrites from their apical dendrite (Price and Powell, 1970). While this neurite bears spines, it also exhibits many properties of axons for which Na_v_ play a particularly important functional role. GC dendrites are known to dispose of Na_v_-dependent backpropagation that can be blocked by TTX (Egger et al., 2003), and show dendritic Na^+^ spikelets both *in vitro* and *in vivo* (Mori and Takagi, 1978; Wellis and Scott, 1990; Luo and Katz, 2001; Pinato and Midtgaard, 2005; Zelles et al., 2006; Labarrera et al., 2013; Burton and Urban, 2015). There is also accumulating evidence for an essential role of Na_v_ in reciprocal processing at the GC spine, where their local activation triggered by AMPAR-mediated EPSPs can further depolarize the spine to activate the classical voltage-gated presynaptic N/P/Q Ca^2+^ channels (Bywalez et al., 2015). Thus it seems likely that GC APs evoke robust Na^+^ signals detectable by Na^+^ imaging in both apical GC dendrites and spines.

Based on this assumption, we chose to probe dendritic Na^+^ signaling in MCs and GCs using both single APs and short AP trains at frequencies of 50 and 80 Hz, which correspond to the maximal frequencies of network oscillations in the olfactory bulb that dendrodendritic MC-GC synapses are involved in (slow and fast gamma range, e.g., Manabe and Mori, 2013). Our main aim was to further elucidate the active properties of GC apical dendrites via direct observation and characterization of AP-evoked Na^+^ signals.

## Materials and Methods

### Animals and Slice Preparation

Rats were decapitated under deep anesthesia with isoflurane according to the stipulations of the German law governing animal welfare (Tierschutzgesetz) and according to the EU directive 2010/63/EU, as approved by the Bavarian state government (Regierung von Oberbayern). Brains were removed and horizontal olfactory bulb brain slices (300 μm thick) were prepared of juvenile wild-type (Wistar) of either sex (postnatal days 11 – 18). The slices were incubated in artificial cerebrospinal fluid (ACSF, composition: 125 mM NaCl, 26 mM NaHCO_3_, 1.25 mM NaH_2_PO_4_, 20 mM glucose, 2.5 mM KCl, 1 mM MgCl_2_, and 2 mM CaCl_2_) infused with carbogen gas (95% O_2_, 5% CO_2_) in a heated water bath at 33°C for 30 min and then kept at room temperature (22°C) until experimentation.

### Two-Photon Imaging and Electrophysiology

Fluorescence was recorded by two-photon laser scanning microscopy (TPLSM) on a Femto-2D microscope (Femtonics, Budapest, HU), equipped with a tunable, Verdi-pumped Ti:Sa laser (Chameleon Ultra I, Coherent, Glasgow, Scotland). The microscope was equipped with a 60× Nikon Fluor water-immersion objective (NA 1.0; Nikon Instruments, Melville, NY, USA), and controlled by MES v4.5 software (Femtonics).

GC and MC somata were patched in whole-cell mode with patch pipettes (resistance 4–5 MOhm), filled with an intracellular saline (composition: 130 mM K-methylsulfate, 10 mM HEPES, 4 mM MgCl_2_, 2.5 mM Na_2_ATP, 0.4 mM NaGTP, 10 mM Na-phosphocreatine, 2 mM ascorbate, 1 mM SBFI (sodium-binding benzofuran isophthalate, Teflabs, Austin, TX and Molecular Probes, Eugene, OR, USA)) at pH 7.3. Electrophysiological recordings were made with an EPC-10 amplifier using Patchmaster software (both HEKA Elektronik, Lambrecht/Pfalz, Germany).

All experiments were performed at room temperature (22°C). The patched MCs and GCs were held in current-clamp mode near their resting potential of -60 and -70 mV, respectively, and the access resistance was monitored. Polarizing step pulses for 500 ms each (first step -90 pA, increased by +50 pA for 10 steps, ending at +180 pA) were applied in order to identify the targeted cell type via its firing pattern. During Na^+^ imaging experiments, cells with a holding current value above -25 pA for GCs and above -50 pA for MCs were rejected.

For Na^+^ imaging, the excitation wavelength of the laser was set to 800 nm and cells were loaded with 1 mM SBFI via the patch pipette. After sufficient filling of the dendritic tree (at least 15 min past establishment of the whole-cell configuration), structures of interest were imaged in free line-scanning mode with a temporal resolution of ∼0.5 ms. At a given dendritic location, several consecutive focal line-scans were recorded during either somatically evoked single action potentials (sAPs) (evoked by an injected current step of 1000 pA) or action potential trains (20 steps at 50 Hz or 80 Hz). To ensure reliable AP induction, current steps had a duration of 5 ms. The duration of the scanning was set to 3.5–5.0 s depending on the decay time course of the Na^+^ transients. Transients recorded sequentially at the same location were averaged and smoothed *post hoc*.

*Post hoc* data analysis was performed using custom macros written in IGOR Pro 7 (Wavemetrics, Lake Oswego, OR, USA) and OriginPro (OriginLab Corporation, Northampton, MA, USA). To correct for dye bleaching, Na^+^ imaging was periodically performed without stimulation, during phases of low postsynaptic spontaneous activity. An exponential decay curve was fitted to these data and then subtracted from the traces during which a stimulation was performed.

Dendritic Na^+^ transients, reflected by decreases in fluorescence emission Δ*F*/*F*, were analyzed relative to the resting fluorescence F_0_, with their decay measured in terms of half-duration τ_1/2_ (Egger et al., 2003). τ_1/2_ values were capped at 3 s, because a higher value could not be reliably extrapolated. Rise times of transients were analyzed in terms of the time interval from 20 to 80% of the maximal Δ*F*/*F* amplitude.

At the end of individual experiments, the neurites were imaged in a z-stack both in the fluorescence and the infrared channel of the TPLSM. Distance measurements were performed using Fiji’s Neurite Simple Tracer plugin (Longair et al., 2011) from z-stack scans of patched cells. The first initial branching of the neurite from the soma was selected as the starting point for the measurement. From that point on, the dendrite was traced in 3D up to the imaged scan line or its first branchpoint and the length of this tracing was determined. The distance of GC somata from the lower border of the MCL was determined in scans imaged in the infrared channel of the TPLSM.

### Simulations

The NEURON simulation tool (Release 7.4, Carnevale and Hines, 2006) was used with Python (Hines et al., 2009) to construct a simple compartmental model of the GC apical dendrite for a simulation of Na^+^ entry. Geometric dimensions were based on previous data (mean diameter of distal GC dendrite 1.2 μm, Egger and Stroh, 2009) and on two-photon fluorescence scans of the neurons in this data set (diameter of proximal segment for decreasing cells 2.7 ± 0.2 μm, *n* = 6 GCs; for increasing cells 2.7 ± 0.4 μm, *n* = 4). The model consists of 11 connected cylindrical compartments, with the first representing the soma (10 μm diameter and length) and the 10 others a dendrite with linear taper from 2.7 to 1.2 μm or 2.0 to 1.2 μm depending on the cell subtype and 200 μm total dendritic length.

In addition to the passive parameters (*R*_m_ = 5000 Ωcm^2^, *R*_i_ = 100 Ωcm, *C*_m_ = 1 μF/cm^2^) the model was equipped with two active conductances for Na^+^ and K^+^ channels (g_Na_ and fast g_K_ as in Zylbertal et al., 2015), and radial and longitudinal diffusion for Na^+^ (as in Zylbertal et al., 2015). The stimulation was implemented as the experimental 50 Hz train of 1000 pA current injections. The basal sodium concentration was set to 15 mM.

Core assumptions of the model were

(i) faithful propagation of AP trains into proximal and distal dendrite which is in part supported by Ca^2+^ imaging data (Egger et al., 2003, but see Discussion).
(ii) g_Na_ and g_K_ models as above, which are unlikely to perfectly capture physiological Na^+^ entry itself; both the precise channel kinetics in general and the detailed Na_v_/K_v_ subtype composition in GC dendrites are not known, therefore we used the model to estimate current densities rather than densities of fictive channels.
(iii) a fixed ratio of g_Na_/g_K_ throughout all compartments (as in Zylbertal et al., 2015).
(iv) linear gradients in g_Na_ density along the dendrite.

The source code for this model is available from ModelDB^1^ as model entry 225086.

### Statistical Analysis

Statistics were performed with the VassarStats online software^2^. The non-parametric Mann–Whitney test was used for all comparisons between two groups except for paired data [e.g., for (Δ*F*/*F*)_50Hz_ and (Δ*F*/*F*)_80Hz_ at the same location, or spines and their adjacent dendrite] where the Wilcoxon pair test was used. For correlations, a linear regression analysis was utilized to determine *r*-values. A cluster analysis of GC properties was performed using the online tool ClustVis (Metsalu and Vilo, 2015), after normalizing the individual parameter values for each property onto the range 0 – 1. Data are presented as mean values of parameters ± standard deviation (SD), unless indicated otherwise.

## Results

### Calibration and General Approach

To allow for the conversion of fluorescence decrements detected by our TPLSM into absolute changes in intracellular [Na^+^], we calibrated SBFI fluorescence signals from cells in olfactory bulb slices following a procedure described earlier (Rose et al., 1999; Meier et al., 2006). Such an *in situ* calibration is required because the properties of SBFI change in intracellular environments, most likely because of increased viscosity. For example, SBFI fluorescence absorption spectra show a significant blue shift when loaded inside cells (e.g., Langer and Rose, 2009). In addition, its K^+^ sensitivity is significantly decreased as compared to *in vitro* calibrations (e.g., Rose and Ransom, 1996; Meier et al., 2006).

**FIGURE 1:**
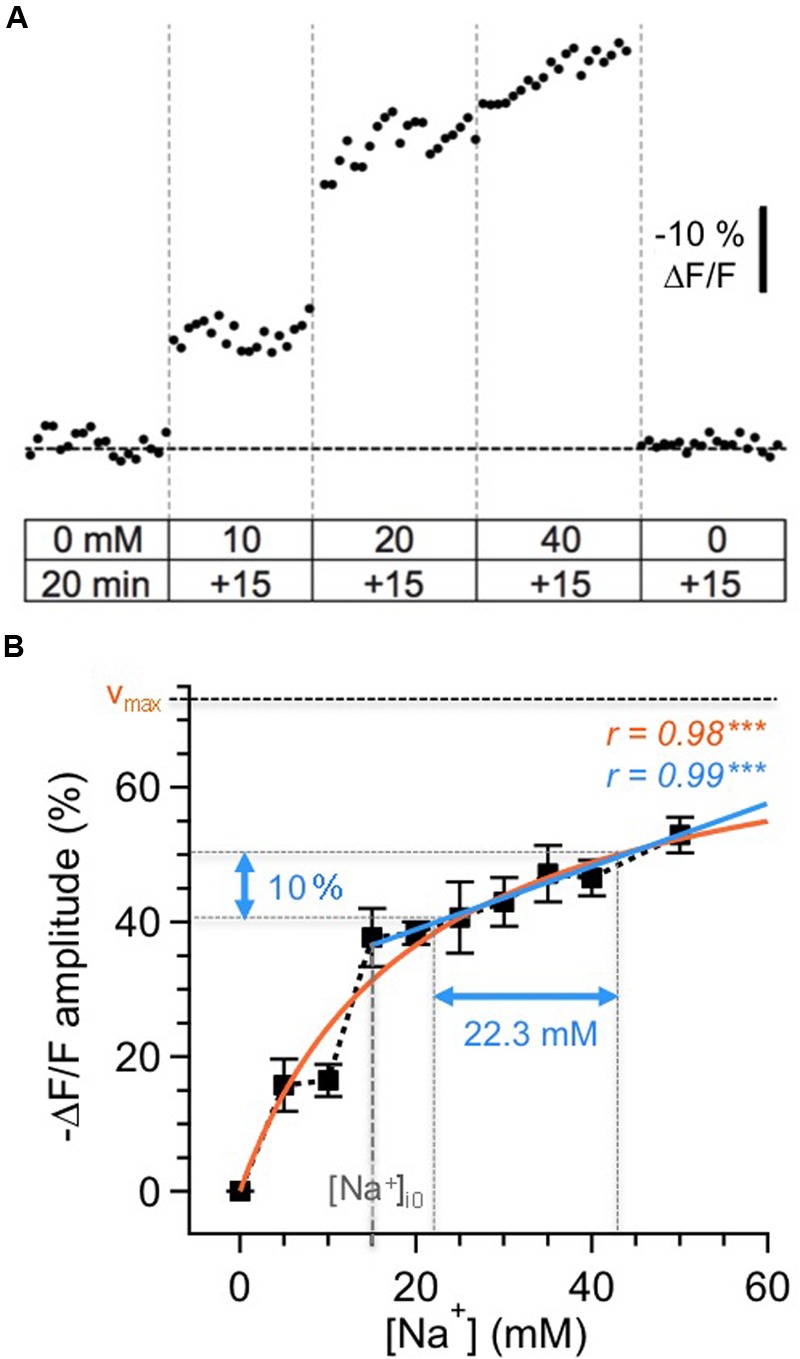
**Calibration of SBFI fluorescence. (A)** Calibration of the Na^+^ sensitivity of SBFI in GC somata in acute tissue slices of the olfactory bulb. Cells were bolus-loaded with the AM-form of SBFI and afterwards subjected to different calibration solutions containing ionophores, the Na^+^/K^+^-ATPase inhibitor ouabain and different Na^+^ concentrations as indicated. Stepwise changes in the extracellular [Na^+^] from 0 to 50 mM for 15 min each and back caused stepwise changes in the fluorescence of SBFI. The trace represents an average from 12 cells obtained in one experiment. **(B)** Relationship between changes in the fluorescence of SBFI and intracellular sodium concentration [Na^+^]_i_, normalized to the fluorescence level in Na^+^-free saline (0 mM). Shown are mean values ± SEM (*n* = 54 cells, five slices, three animals). The orange line represents a Michaelis–Menten fit of all the data (*v*_max_ = 73.5%, *K*_D_ = 20.2 mM). The blue line represents a linear fit of the data obtained between 15 and 50 mM [Na^+^]_i_. Within this range, a 10% change in fluorescence emission of SBFI corresponds to a change of 22.3 mM in [Na^+^]_i_.

For calibration, cells were loaded by bolus injection with the membrane-permeable form of SBFI (SBFI-AM) into the GC layer and MC layer (MCL) as reported earlier for other brain regions (Langer and Rose, 2009; Karus et al., 2015). Next, equilibration of intra- and extracellular Na^+^ concentration was achieved by perfusing SBFI-loaded slices with calibration solutions containing ionophores (3 μM gramicidin D and 10 μM monensin) and the Na^+^/K^+^-ATPase blocker ouabain (100 μM). Slices were then perfused with calibration salines containing different Na^+^ concentrations and resulting changes in SBFI fluorescence were recorded (*n* = 54 cells; five slices, three animals; cf. **Figures 1A,B**). As reported earlier (Rose et al., 1999; Meier et al., 2006; Mondragao et al., 2016), changes in relative fluorescence levels were highly correlated to changes in [Na^+^]_i_ concentrations, with increasing [Na^+^] causing a decrease in fluorescence emission of SBFI.

The changes in relative fluorescence (*F*/*F*_o_) against [Na^+^]_i_ could be fit by a Michaelis–Menten relationship (*r* = 0.98), revealing an apparent *K*_D_ of 20.2 mM and a maximal change in fluorescence of *v*_max_ = 73.5%. This observation indicates that SBFI fluorescence starts to saturate at levels well beyond 50 mM [Na^+^]_i_, and is thus well suited to report [Na^+^]_i_ changes within the physiological range.

Pipette [Na^+^] was 15 mM in our experiments. Under the assumption that this concentration also represented the baseline [Na^+^]_i_ of patch-clamped neurons, a linear correlation was extrapolated for [Na^+^]_i_ between 15 and 50 mM (*r* = 0.99, *P* < 0.001), with -10% Δ*F*/*F* corresponding to an increase Δ[Na^+^]_i_ of 22.3 mM. Because our study only reports increases in [Na^+^]_i_ rising from the assumed baseline of 15 mM, we chose this simple linear correlation (the *r*-value of which was even slightly higher than that of the Michaelis–Menten plot), to convert changes in SBFI fluorescence to changes in [Na^+^]_i_. Importantly, and in contrast to the situation if a Michaelis–Menten relationship is applied, this linearization renders data conversion independent from the actual baseline levels, which – in active neurons undergoing axonal and dendritic sodium influx – might be higher that the presumed baseline of 15 mM.

**FIGURE 2:**
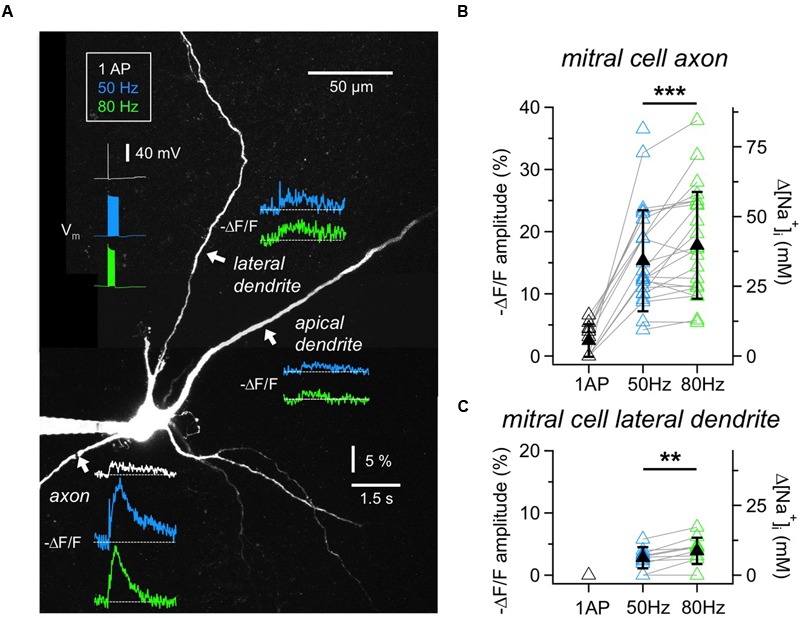
**Na^+^ signals in MC axons and dendrites. (A)** Two-photon scan of a representative MC filled with 1 mM SBFI. Averaged voltage traces of the three stimulation protocols (1 AP, 20 APs at 50 or 80 Hz, train duration 400 ms or 250 ms) as recorded from the soma are shown in the upper left. Averaged fluorescence transients were imaged at the locations indicated with white arrows. Fluorescence changes in response to single APs could only be detected in the axon. **(B)** Cumulative Δ*F*/*F* amplitude data for MC axons (*n* = 26 cells) and **(C)** lateral dendrites (*n* = 12 cells). Open symbols: individual data, solid black symbols: averaged data ± SD. Data recorded from the same location are indicated by gray connecting lines. ^∗∗^*P* < 0.01; ^∗∗∗^*P* < 0.005.

Throughout the remainder of this study, neurons were filled with 1 mM of SBFI in the whole-cell configuration and stimulated with somatic current injections to generate either single APs or trains of 20 APs at 50 Hz or 80 Hz (train duration 400 ms and 250 ms, respectively; see **Figure 2A**).

### Mitral Cell Axons and Dendrites

To establish measurements of Na^+^ transients, we first imaged the initial segment of MC axons (**Figures 2A,B**). Single APs elicited detectable axonal Δ*F*/*F* changes in 6 out of 11 MCs tested (responding axons: mean (Δ*F*/*F*)_AP_ = 4.7 ± 1.2%, corresponding to roughly 10 mM Δ[Na^+^]_i_). In our set of experiments the detection threshold was ∼2% Δ*F*/*F*, corresponding to 4 mM Δ[Na^+^]_i_. All axons showed substantial fluorescence changes in response to trains of 50 Hz (*n* = 25 cells, mean (Δ*F*/*F*)_50Hz_ = 15.3 ± 8.1%, corresponding to 34 mM Δ[Na^+^]_i_) and 80 Hz (*n* = 24, mean (Δ*F*/*F*)_80Hz_ = 17.8 ± 8.6%, corresponding to 40 mM Δ[Na^+^]_i_), at a mean distance from the soma of 34 ± 14 μm. We chose to image the axon not too close to the MC soma since we observed that more proximal measurements yielded smaller Δ*F*/*F* measurements (*n* = 3 MCs). While peak amplitudes of Na^+^ transients measured at the same axonal location increased significantly at 80 Hz as compared to the 50 Hz stimulation (*n* = 22, mean ratio_80/50_ = 1.25 ± 0.33, *P* < 0.005, Wilcoxon test, **Figure 2B**), the transients decayed with indistinguishable half-durations of τ_1/2_50Hz_= 0.61 ± 0.45 s and τ_1/2_80Hz_= 0.60 ± 0.51 s (not shown).

We also investigated Na^+^ transients in MC lateral dendrites because of their involvement in the reciprocal microcircuits with GCs (**Figures 2A,C**). The measurement locations were on average 66 ± 34 μm distal from the soma (*n* = 12). Single APs did not evoke detectable changes in SBFI fluorescence. Trains of APs induced small, but reliable fluorescence changes in the majority of cells (50 Hz: *n* = 10 out of 12 cells, mean (Δ*F*/*F*)_50Hz_ = 3.2 ± 1.1% or 7 mM Δ[Na^+^]_i_; 80 Hz: *n* = 9 out of 10, mean (Δ*F*/*F*)_80Hz_ = 4.4 ± 1.7% or 10 mM Δ[Na^+^]_i_). Again, peak amplitudes significantly increased between 50 and 80 Hz at the same location (*n* = 8, mean ratio_80/50_ = 1.43 ± 0.35, *P* < 0.01, **Figure 2C**). The transients decayed with similar half-durations of τ_1/2_50Hz_= 0.91 ± 0.64 s and τ_1/2_80Hz_= 0.88 ± 0.57 s. Because of the small size of transients we did not explore the propagation of signals along the lateral dendrites.

Finally, in MC proximal apical dendrites (*n* = 8 cells, mean distance from the soma 58 ± 44 μm) fluorescence changes in response to trains were barely detectable, on the order of 4 mM Δ[Na^+^]_i_ (**Figure 2A**; 50 Hz: *n* = 2 out of 8 cells tested, mean (Δ*F*/*F*)_50Hz_ = 2.0 ± 0.7%; 80 Hz: *n* = 4 out of 7, mean (Δ*F*/*F*) _80Hz_= 2.2 ± 0.5%), not warranting further investigation.

### Granule Cell Apical Dendrites

**FIGURE 3:**
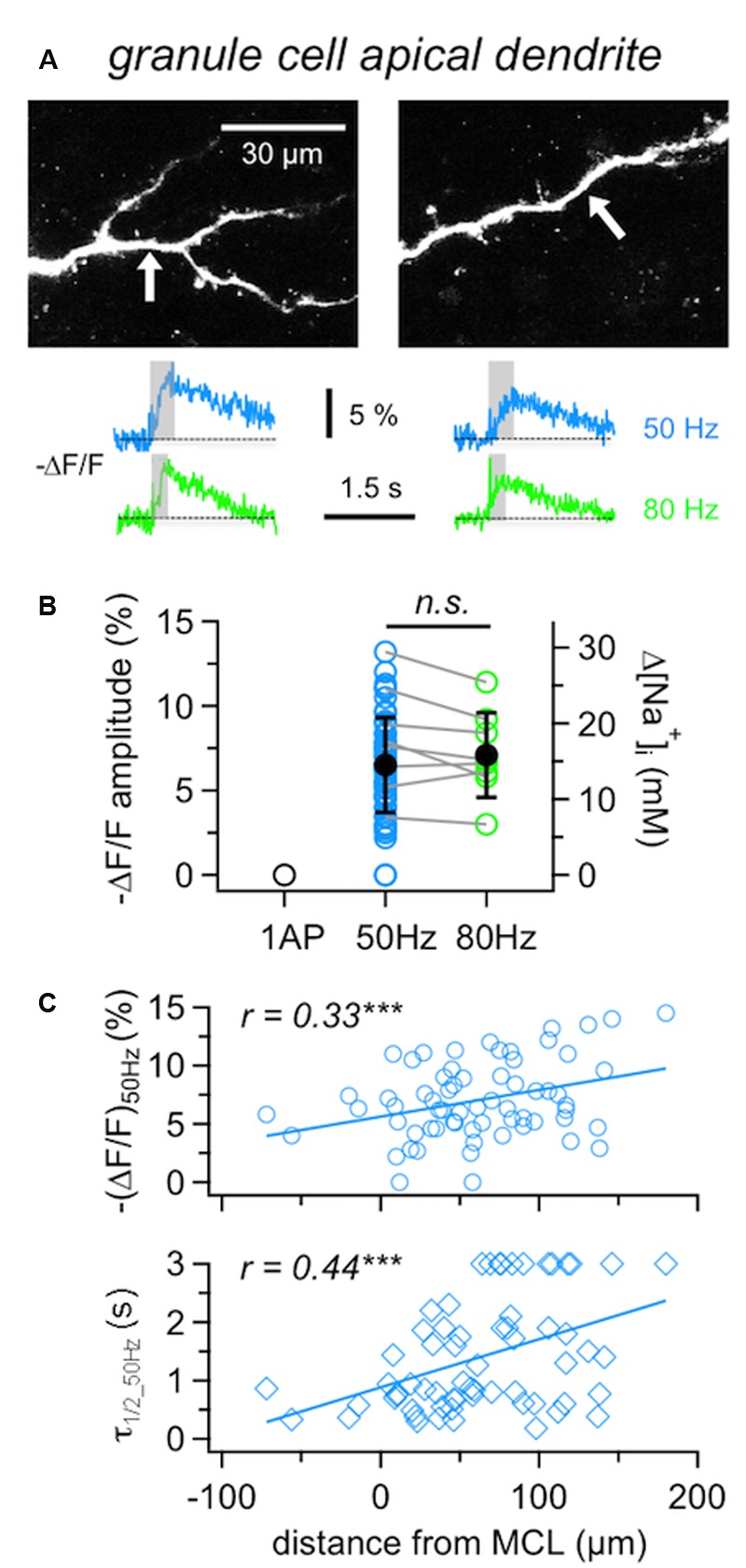
**Na^+^ signals in GC apical dendrites. (A)** Representative two-photon scans of apical dendritic segments from two GCs filled with 1 mM SBFI. The averaged fluorescence transients shown below were imaged at the locations indicated with white arrows. Train durations are indicated by the gray boxes. **(B)** Cumulative Δ*F*/*F* amplitude data for GC apical dendrites (*n* = 62 locations in 36 cells). Open symbols: individual data, solid black symbols: averaged data ± SD. Data where responses to both 50 and 80 Hz AP trains were recorded from the same location are indicated by gray connecting lines (*n* = 8 cells). **(C)** Cumulative data for responses to 50 Hz trains recorded from GC apical dendrites plotted versus the distance of the recording location from the lower border of the MC layer. Top panel: Δ*F*/*F* amplitude, bottom panel: Δ*F*/*F* half duration τ_1/2_. Lines: Linear fits of the data. Weak positive correlations were observed for both the amplitude and the half duration. ^∗∗∗^*P* < 0.005.

In GC apical dendrites, responses to single APs could not be resolved (tested in five cells), while fluorescence changes upon 50 Hz trains could be detected in almost all locations tested (in 62 out of 65 locations in 37 GCs; mean (Δ*F*/*F*)_50Hz_ = 6.9 ± 3.3%, corresponding to 15 mM Δ[Na^+^]_i_; **Figure 3**), at an average distance from the soma of 75 ± 42 μm.

Compared to the MCs’ responses to 50 Hz AP trains, dendritic GC Na^+^ transients were significantly smaller and slower than MC axonal transients ((Δ*F*/*F*)_50Hz_: *P* < 0.0001, τ_1/2_50Hz_: *P* < 0.0005) and significantly larger than MC lateral dendrite transients (*P* < 0.001) with no detectable difference in τ_1/2_50Hz_.

Since 80 Hz trains did not yield significantly increased transients in GCs compared to 50 Hz trains (**Figure 3B**, *n* = 8 tested cells, (Δ*F*/*F*)_50Hz_ = 8.0 ± 3.1% vs. (Δ*F*/*F*)_80Hz_ = 7.2 ± 2.5%, mean ratio_80/50_ = 0.92 ± 0.13, n.s.), we restricted experiments to 50 Hz in order to be able to conduct measurements in several dendritic locations in the same cell.

It has been reported before that GC Ca^2+^ signals in response to single APs increase with distance from soma and show a plateau within the EPL (Egger et al., 2003). GC Na^+^ signals in response to 50 Hz trains showed similar but less pronounced effects, since (Δ*F*/*F*)_50Hz_ amplitudes were weakly positively correlated to the distance of the measurement location from the MCL border (**Figure 3C**, *r* = 0.33, *P* < 0.005; see also below).

**FIGURE 4:**
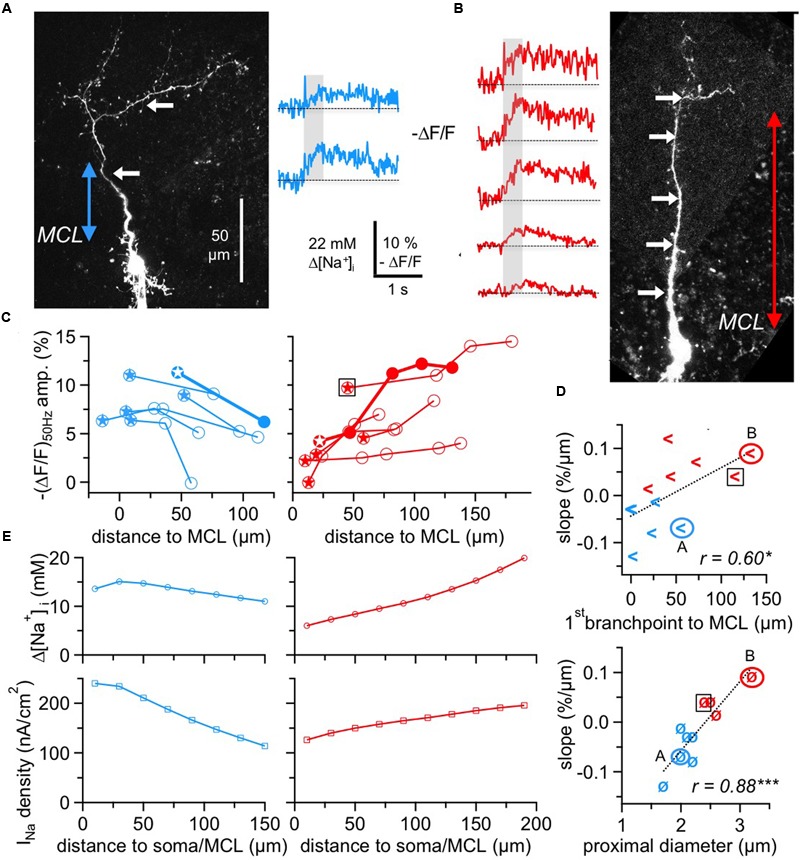
**Two subtypes of GC Na^+^ signals versus dendritic distance. (A,B)** Two-photon scans of GCs representative for the decreasing subtype (left panel, blue transients) and for the increasing subtype (right panel, red transients) with averaged fluorescence transients in response to 50 Hz AP trains imaged at the locations indicated by the white arrows. The colored arrows in the scans indicate the distance from the lower MCL border as established in the infrared scan to the first branch point of the apical dendrite. Train durations are indicated by the gray boxes. **(C)** Cumulative plots of (Δ*F*/*F*)_50Hz_ amplitudes vs. distance from MCL border for the decreasing (left panel, *n* = 6 cells) and increasing (right panel, *n* = 6 cells) subtype, respectively. All data points belonging to the same GC are interconnected by lines. The data of the cells shown in **(A,B)** are indicated by solid symbols and thick connecting lines. Stars indicate the most proximal measurement for each cell. The open black square denotes the GC that was not clustered with the other cells with positive slope (see Results). **(D)** Top panel: Plot of the cell-wise slope of (Δ*F*/*F*)_50Hz_/distance from MCL versus the distance of the respective GC’s first branch point from MCL. Bottom panel: Plot of the cell-wise slope of (Δ*F*/*F*)_50Hz_/distance from MCL versus the diameter of the proximal dendritic segment. Again, the open black squares denote the GC that was not clustered with the other cells with positive slope. **(E)** Simulation of Δ[Na^+^]_i_ (top panels) and the associated Na_v_ current density I_Na_ (bottom panels) in a simple compartmental model of the apical dendrite, with an initial diameter of 2.0 μm for the decreasing and of 2.7 μm for the increasing cell type, both tapering linearly to 1.2 μm over 200 μm. I_Na_ was adjusted to match the average experimental [Na^+^]_i_ for the respective cell type from panel C above (see Materials and Methods). ^∗^*P* < 0.05; ^∗∗∗^*P* < 0.005.

Granule cell dendritic Na^+^ transients (Δ*F*/*F*)_50Hz_ decayed with a mean half duration of τ_1/2_50Hz_= 1.02 ± 0.59 s (*n* = 54 locations). Several distal locations showed half durations of ≥3 s (*n* = 12), which could not be properly determined due to the limited scan time and/or noise (Methods) and thus were not included in the above mean value. Overall, a correlation with distance was observed, with a clear trend for slow transients to occur in the distal parts of the dendrites (**Figure 3C**, *r* = 0.43, *P* < 0.001; see also GC in **Figure 4B**). The average rise time of (Δ*F*/*F*)_50Hz_ was *t*_rise_ = 0.30 ± 0.12 s (*n* = 61 locations) and uncorrelated with distance from the soma (*r* = 0.24, *P* = 0.06), indicating that later APs within 50 Hz trains did not fail to invade distal parts of the dendrite (data not shown).

While the pooled data across all GCs and locations (**Figure 3C**) suggested a rather weak influence of dendritic location on Na^+^ peak amplitude, analysis of distance versus (Δ*F*/*F*)_50Hz_ amplitude within individual GCs revealed a more refined picture (**Figure 4**). Here, we included all GCs with at least two dendritic measurement locations at a distance of ≥ 50 μm between the first and last location.

We observed two different behaviors, with a subset of GCs showing a consistent decrease (*n* = 6 of 12 cells) and the remainder (*n* = 6 cells) a consistent increase of peak amplitudes, as shown in **Figure 4C**. The average linear slopes of each subtype’s pattern were similar except for their sign (-0.07 ± 0.04% Δ*F*/*F* per μm vs. 0.06 ± 0.04% Δ*F*/*F* per μm). Interestingly, all the GCs with the increasing pattern except for one showed smaller Na^+^ transients within the most proximal apical dendrite measurements (with respect to the MCL; < 5% Δ*F*/*F* or 11 mM Δ[Na^+^]_i_) whereas all GCs with the decreasing pattern showed larger proximal Na^+^ transients (> 5% Δ*F*/*F*; *P* < 0.025; **Figure 4C**).

Since absolute concentration changes are tightly linked to dendritic SVRs and thus to dendritic morphology, we explored possible correlations between morphology and the observed Na^+^ transient patterns. Because the morphologies of individual GCs could not always be fully recovered at the end of experiments, our analysis was restricted to the parameters that were available for all or most GCs. While we could not detect any significant link to branching itself, i.e., a systematic drop or increase in (Δ*F*/*F*)_50Hz_ for measurement locations in front of versus beyond individual branch points, there were correlations between the slope of Δ*F*/*F* per μm and the distance of the first branchpoint from the MCL and also between the slope and the diameter of the proximal dendrite (**Figure 4D**, *r* = 0.60, *P* < 0.025 and *r* = 0.89, *P* < 0.005). The location of the first branchpoint is indicative of the anatomical subtype of GC: small, compact GCs like the one in **Figure 4A** with early branching preferentially innervate the deep EPL, whereas GCs with dendrites innervating mostly higher levels of the EPL also usually show more distal first branch points (Mori et al., 1983; Orona et al., 1983; see Discussion). GCs with negative slope had their first branchpoint on average 20 ± 22 μm above the MCL, whereas the first branchpoints of GCs with positive slope were located at 70 ± 45 μm (*P* < 0.025).

A cluster analysis of GCs with respect to the four parameters (slope of transients, amplitude of most proximal Δ*F*/*F* measurement, distance of first branch point and diameter of proximal dendrite, see Materials and Methods) indeed resulted in a clustering into two groups according to slope sign, except for the increasing cell with the high initial Δ*F*/*F* marked in the left panel of **Figure 4C**. However, since this data point is already rather remote from the MCL, a more proximal measurement in the same cell might have been of considerably smaller amplitude.

To provide a qualitative explanation of the observed GC subtype behavior, we used a simple compartmental model (see Materials and Methods, **Figure 4E**) in which we varied the Na^+^ current density along the dendrite and matched its values to the observed Δ[Na^+^]_i_. Based on the key assumption that AP trains faithfully propagate into the dendrite, these simulations yielded a negative gradient for the decreasing pattern that would be about two times stronger than the positive gradient for the increasing pattern. This difference as well as the plateau in the proximal part of the decreasing cell pattern can be explained by the effect of tapering, which by itself creates an increase in Δ[Na^+^]_i_ with distance because of the increased SVR. The model was robust with respect to branching and effects of Na^+^ diffusion, both in line with our data.

### Granule Cell Large Spines and Adjacent Dendritic Shafts

**FIGURE 5:**
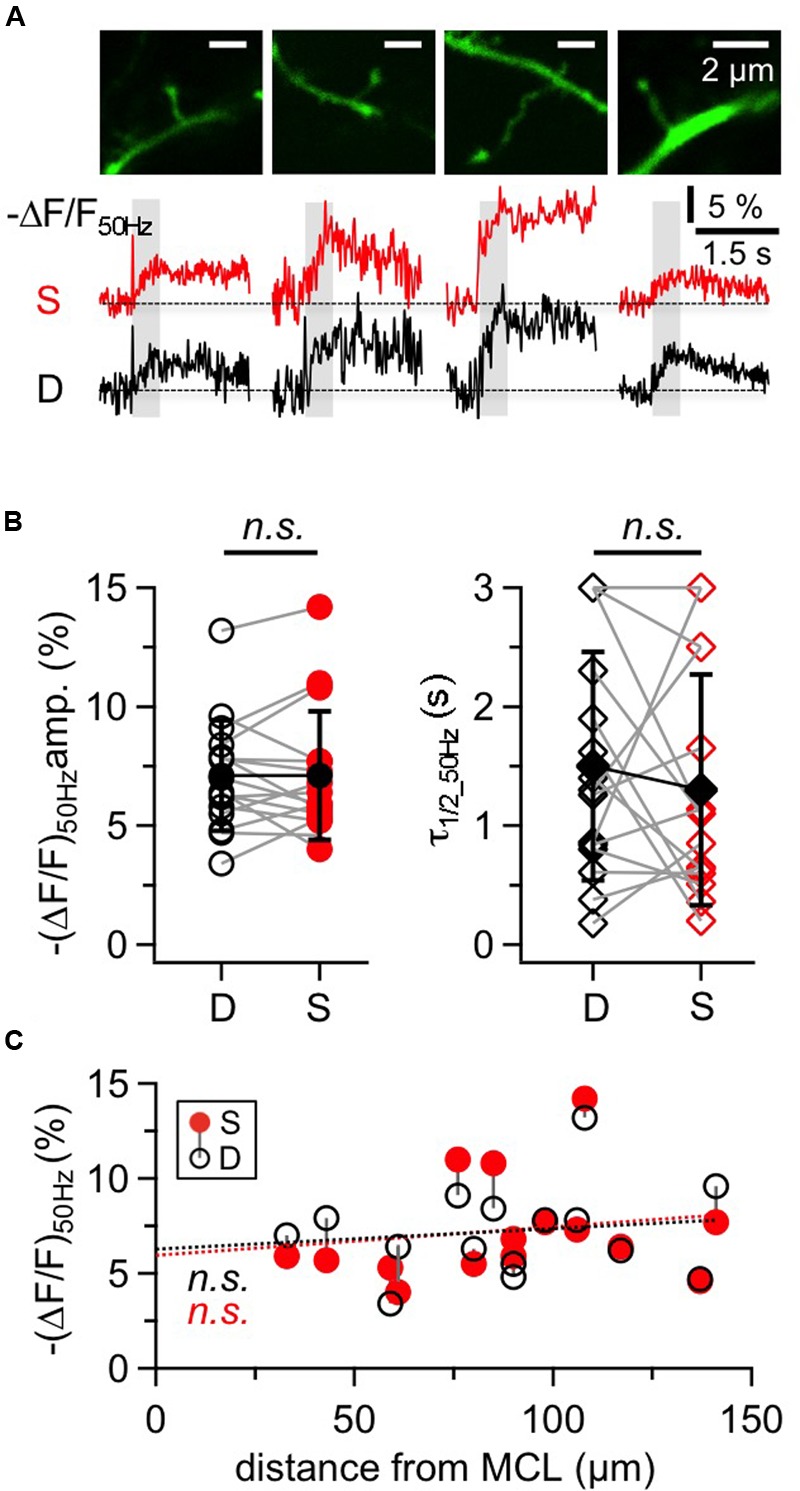
**Na^+^ signals in large GC spines and their adjacent dendrites. (A)** Two-photon scans of four representative large GC spines with averaged fluorescence transients in response to 50 Hz trains imaged at the spine heads (S, red traces) and the adjacent dendrites (D, black traces). Train durations are indicated by the gray rectangles. **(B)** Cumulative data for all spine-dendrite pairs for (Δ*F*/*F*)_50Hz_ amplitudes and half durations. (*n* = 16 pairs). Open symbols: individual data, solid black symbols: averaged data ± SD. Data from the same spine-dendrite pair are interconnected. Because Δ*F*/*F* in more than one location showed half durations ≥3 s, the respective data are accumulated in the 3 s data points and more than one pair can be connected to these points. **(C)** Cumulative data for all spine-dendrite pairs for (Δ*F*/*F*)_50Hz_ amplitudes versus their distance from the MCL border. There is no significant trend, and the linear fits for spine and dendrite data each (red and black dotted line) are highly similar.

The large GC spines on the apical dendrite are known to contain reciprocal synapses and to exhibit voluminous spine heads and long spine necks (Woolf et al., 1991). **Figure 5A** shows representative responses to 50 Hz trains within putative reciprocal GC spines and their adjacent dendritic shafts. The 16 spines in our sample were located on average 98 ± 31 μm away from the soma and responded with a mean (Δ*F*/*F*)_50 Hz_ = 7.1 ± 2.7% corresponding to 16 mM Δ[Na^+^]_i_. Again, the decay of GC spine Na^+^ transients was mostly on the order of 1 s with a mean half duration τ_1/2_50 Hz_ = 1.10 ± 0.76 s (*n* = 12 spines) with a subset of four spines showing half durations of ≥ 3 s that could not be precisely measured as described above for the GC dendrite.

Compared to the adjacent dendrite, there were no consistent differences between the transients of a given spine and its dendrite with respect to amplitude, half duration and rise time, as shown in **Figures 5A,B** (ratio S/D Δ*F*/*F*_50 Hz_ = 1.02 ± 0.24, *n* = 16, *P* = 0.76; ratio S/D τ_1/2_50 Hz_ = 1.29 ± 1.27, *n* = 15, *P* = 0.50; ratio S/D t_rise_50 Hz_ = 1.09 ± 0.44, *n* = 14, *P* = 0.91). Spine transients as a whole set also showed no correlation with distance from the MCL, for both amplitudes (**Figure 5C**) and half durations (not shown).

## Discussion

### Technical Aspects: Suitability of Na^+^ Imaging to Explore MC and GC Function

In this study, we have established two-photon Na^+^ imaging with the fluorescent indicator dye SBFI to explore propagation of action potentials based on Na^+^ influx through voltage-gated channels in mitral and GC neurites. Compared to commonly used chemical calcium indicators such as Fura-2 or Oregon-Green-Bapta-1, SBFI has a low quantum efficiency and exerts a high *K*_D_ (Schreiner and Rose, 2012). Moreover, both relative changes in [Na^+^]_i_ over baseline and relative changes in dye emission are rather small during physiological activity, necessitating the use of high dye concentrations to obtain an acceptable signal-to-noise-ratio (Rose et al., 1999). Importantly, and again in contrast to high-affinity Ca^2+^ indicator dyes (Neher and Augustine, 1992; Maravall et al., 2000), SBFI does not buffer Na^+^ and, therefore, does not distort Na^+^ signals even at a dye concentration of 1 mM as used in the present study (Sabatini et al., 2001; Mondragao et al., 2016).

Similar to earlier reports (e.g., Mondragao et al., 2016), our *in situ* calibrations revealed an acceptable linear correlation between changes in SBFI emission (Δ*F*/*F*) and changes in [Na^+^]_i_ between 15 and 40–50 mM [Na^+^]_i_. This enabled a calculation of absolute changes in [Na^+^]_i_ without the necessity of knowing baseline [Na^+^]_i_. Such quantitative measurements with two-photon microscopes are far more tedious for Ca^2+^ concentration changes, where basal and maximal fluorescence has to be established in every cell of interest (e.g., Maravall et al., 2000; Egger and Stroh, 2009). Taken together, these considerations illustrate that SBFI is an indicator well-suited for quantitative measurement of activity-induced [Na^+^]_i_ transients.

Large [Na^+^]_i_ transients were found in MC axons following somatic current injection and induction of APs. Even single APs evoked detectable Δ[Na^+^]_i_ in half of the axons, amounting to an average increase by 10 mM, while trains of APs resulted in Δ[Na^+^]_i_ transients by more than 30 mM. Activity-related [Na^+^]_i_ signals in this range have also been reported from dendrites and spines of CA1 pyramidal neurons in response to suprathreshold synaptic stimulation and opening of ionotropic glutamate receptors (Rose and Konnerth, 2001). Interestingly, peak amplitudes were largest at a distance of ∼35 μm from the soma. While we have not investigated this observation in greater detail, it indicates that the spike initiation zone is not directly located at the cell body but at some distance from it, as demonstrated before based on Na^+^ imaging or patch recordings from axon initial segments, e.g., in cortical pyramidal neurons (Kole et al., 2008; Hu et al., 2009; Fleidervish et al., 2010), cochlear interneurons (Bender and Trussell, 2009) and dentate gyrus GCs (Scott et al., 2014). Since in a few MCs the train-evoked transients reached amplitudes beyond the linear regime of calibration, we would recommend to use shorter AP trains for future studies of MC axonal Na^+^ signaling.

Action potential-induced axonal Na^+^ transients rapidly decayed with half-durations of ∼0.6 s. This is in the range of decay times determined for layer five pyramidal neurons (∼0.5 s; Fleidervish et al., 2010). The latter study also provided evidence that the decay of axonal Na^+^ transients is largely governed by fast lateral diffusion. The same phenomenon was described for local, glutamate-induced Na^+^ transients in dendrites of CA1 pyramidal neurons, where decay to baseline was dominated by diffusion, resulting in a more than 10-fold higher rate of recovery as compared to extrusion through the Na^+^/K^+^-ATPase (Mondragao et al., 2016).

Na^+^ imaging also enabled detection of Na^+^ APs backpropagating into MC lateral and apical dendrites. In both dendrite types, Na^+^ influx in response to single APs could not be resolved, while trains of 20 APs resulted in small signals as observed earlier (Rose et al., 1999). However, on our TPLSM signals from both compartments were close to the detection limit, especially in the apical dendrite. Thus we would like to suggest that in MCs SBFI-based Na^+^ imaging will mainly be helpful to explore axonal function or to study activity in the highly excitable apical dendritic tuft (as performed by Zylbertal et al., 2015; not investigated here), whereas it is less suitable for studies of propagation into MC dendrites, which might be better studied via dendritic patching (Chen et al., 1997; Christie and Westbrook, 2003).

In GC apical dendrites we observed substantially larger Na^+^ transients as compared to MC lateral dendrites in response to 50 Hz trains which allowed us to quantitatively compare signals along the dendrite of individual GCs and also across spines and their adjacent dendrites. In general, the observed GC (Δ*F*/*F*)_50Hz_ signals were within the linear regime of our calibration (range 20–45 mM including baseline [Na]_i_ = 15 mM, **Figure 1B**).

### GC Apical Dendrite Na^+^ Signals vs. Ca^2+^ Signals

In an earlier study, we have observed that single sAPs result in Ca^2+^ entry throughout GC apical dendrites (Egger et al., 2003). These sAP-mediated Ca^2+^ signals did not decrease with distance from the soma but rather showed a plateau within the EPL, which we interpreted as a property tied to dendritic transmitter release onto MCs and TCs within the EPL. Here, we found that sAP-train-mediated Na^+^ signals did either rise or decline with distance from the MCL, in a manner that was correlated with morphological features of the respective cells.

Since such differential patterns might have been obscured in the initial Ca^2+^ imaging study due to averaging across cells, we have now performed a cell-wise reanalysis of the Ca^2+^ imaging data set with respect to morphology and slope in (Δ*F*/*F*)_sAP_ but could not reveal any significant correlations between Ca^2+^ transient amplitude changes along the apical GC dendrite and branchpoint location or proximal diameter (*n* = 19 cells; only 4 of these showed negative slopes in (Δ*F*/*F*)_sAP_ in the first place). If we assume that there is little failure of invasion by late APs in the train (as indicated by the lack of a negative correlation between the rise time of (Δ*F*/*F*)_50Hz_ and the distance from soma in the Na^+^ imaging data set, see results), the difference between Na^+^ and Ca^2+^ signaling might be explained by the fact that a substantial fraction of ΔCa^2+^ is carried by low-voltage activated Ca^2+^ channels (Egger et al., 2003; Pinato and Midtgaard, 2005) and thus a slight attenuation or increase in Na^+^ entry with distance might have only a limited effect on the sAP-associated Ca^2+^ signals. Moreover, if the observed Na^+^ signaling patterns can indeed be explained by variations in Na_v_ current density, Ca_v_ current density in turn is unlikely to systematically covary with Na_v_ current density, which also has not been observed in other neuron types (e.g., Magee, 2008). Also, if Na^+^ imaging would simply mirror GC dendritic excitability with respect to Ca^2+^ entry, Na^+^ imaging in GCs would be of little extra use compared to Ca^2+^ imaging since the Na^+^-dye properties do not allow to resolve single APs so far.

To shed light on local correlations between Na^+^ and Ca^2+^ entry, simultaneous dendritic Na^+^ and Ca^2+^ imaging would be required, as performed, e.g., by Jaffe et al. (1992), who observed in CA1 pyramidal cell dendrites sAP-train-mediated Na^+^ and Ca^2+^ signals at distances at least up to 200 μm from the soma. Moreover, they found that distal Na^+^ transients were smaller than proximal signals and that their rise was restricted to the initial phase of sAP trains, probably due to an invasion failure of later APs. Ca^2+^ transients in the same cells were found to mirror this behavior, leading the authors to the conclusion that AP invasion was required for Ca^2+^ influx to occur. In our studies in GCs there was no such indication of propagation failures during trains, probably due to the high excitability of the GC axo-dendrite.

### Functional Aspects: GC Morphology and Dendritic Excitability

The distance of the first branchpoint of the apical GC dendrite from the MCL is indicative of the morphological subtype of GC, as can be gathered from the GC anatomies shown in Mori et al. (1983) and Orona et al. (1983): GCs with early branching preferentially innervate the deep EPL or possibly the entire EPL, while GCs with more distal first branch points innervate mostly higher levels of the EPL. Mori et al. differentiated the subtypes I, II, III depending on the innervated sublayer(s) of the EPL. While our sample of GCs is biased with respect to the distance of the soma from the MCL (all closer than 100 μm), all three subtypes might have been picked up, since the somata of subtype I and II can be found throughout the GCL and subtype III occurs preferentially close to or within the MCL (Mori et al., 1983). In addition, we also might have sampled type V shrub GCs (Merkle et al., 2014; Nagayama et al., 2014). The GCs with positive slope in Δ*F*/*F* would thus correspond mostly to subtype III, projecting into the superficial EPL and thus interacting preferably with TCs. On the other hand, the GCs with decreasing Δ*F*/*F* would belong to subtype II and perhaps also I, interacting rather with MCs. With respect to the GCs in our data set, an unequivocal assignment to these subtypes would require both a complete recovery of GC morphology, including their extent relative to the EPL width, and a more quantitative description of GC morphotypes in the literature.

Nevertheless, our findings for the first time reveal functional differences in active dendritic properties of GCs innervating different sublayers of the EPL. Together with the observation that deep-branching cells start out with larger Na^+^ signals compared to the higher branching cells, these patterns might relate to different Na_v_ channel densities and gradients along the dendrites as implied by our simulations of Na^+^ current densities (**Figure 4E**). Our model also predicts that the increasing pattern is not caused by decreasing dendritic diameters alone (due to the concomitant increase in SVR along the dendrite) but could involve a roughly twofold increase in current density along the dendrite. Similarly diverse types of Na_v_ channel or Na_v_ current distributions have been reported from several other cell types, with a negative gradient of Na_v_1.6 expression in CA1 pyramidal cells (Lorincz and Nusser, 2010), a constant high density in a subtype of hippocampal interneuron (Martina et al., 2000) and an increasing Na^+^ current density in CA3 pyramidal cells (Kim et al., 2012).

In GCs the observed differential patterns might serve to foster AP propagation for GC subtypes with larger dendritic trees or at least might compensate for an increased likelihood of propagation failures because of the extended morphology, while the higher current density in the less wide proximal segment of the smaller cells might support dendrosomatic AP conduction.

If the different subclasses of GCs indeed dispose of different Na_v_ channel densities and different dendritic gradients, these differences could also impact synaptic integration and thus GC subtype interaction with the respective mitral/TC subnetworks via their dendrodendritic output. Recent studies indicate substantial functional differences between MCs and TCs with respect to temporal processing of olfactory stimuli: TCs are firing earlier in the respiration cycle and are thought to encode the detection of the presence of an odor, whereas MCs are firing later and might contribute to difficult odor discriminations (Fukunaga et al., 2012; Igarashi et al., 2012). Moreover, TCs are involved in early onset fast gamma oscillations while MCs contribute to late onset slow gamma oscillations (Manabe and Mori, 2013). Thus the dendritic excitability of their GC partners in the respective subnetworks could be specifically tuned toward these temporal processing requirements. Since increasing cells are preferentially interacting with TCs and are predicted to dispose of a higher current density in their distal dendrites (than the decreasing cells that interact with MCs), it is tempting to speculate that increasing GCs can generate APs (or Na^+^ spikelets, see Introduction) in the distal dendrite and thus lateral output within the GC-TC network more rapidly than decreasing GCs, both in line with a contribution to fast gamma and to fast TC output. Decreasing GCs on the other hand can afford to provide lateral inhibition on a slower time scale, potentially serving to decorrelate MC activity during difficult odor discriminations (Friedrich and Wiechert, 2014).

### GC Spine Na^+^ Signaling

As observed earlier for other cell types such as CA1 pyramidal neurons (Rose et al., 1999), trains of backpropagating APs not only induced Na^+^ signals in GC dendrites, but also in their large spines. GC spine signals did not differ from dendritic Na^+^ signals in respect to their peak amplitude, rise or decay times. These data, therefore, do not allow to draw a clear conclusion about the presence or absence of voltage-gated Na_v_ in spine heads. A recent study in CA1 pyramidal neurons showed that diffusion of Na^+^ ions along dendrites is considerably slower than previously thought (320 μm^2^/s; Mondragao et al., 2016). This value, is, however, still about 20-fold faster than that of buffered diffusion of Ca^2+^ ions (∼15 μm^2^/s; Yuste et al., 2000), indicating that any differences in Na^+^ concentrations between spines and dendrites upon differential Na^+^ influx will be equilibrated rapidly.

On the other hand, there is substantial indirect evidence for the presence of Na_v_ in GC spine heads from Ca^2+^ imaging (Bywalez et al., 2015) where quasi-synaptic stimulation of GC spines via uncaging of glutamate resulted in a TTX-sensitive Ca^2+^ signal that can be explained by the existence of a local post-synaptic Na^+^ spike within the spine head. GC Ca^2+^ signals due to propagation of sAPs or due to synaptically evoked global APs were also found to be very similar in spines and their adjacent dendrites (Egger et al., 2003; Egger, 2008). Moreover, both Ca^2+^ handling within and the SVRs of these two compartments were found to be highly similar (Egger and Stroh, 2009). Taken together, these findings argue for a similar density of Na_v_ channels and low-voltage activated Ca^2+^ channels within GC spines and their adjacent dendrites. To fully validate this conclusion the precise Na^+^ extrusion/diffusion mechanisms in GC dendrites need to be determined.

## Conclusion

In summary, we have shown that Na^+^ imaging can reveal distinct functional aspects of the active GC dendrites that have not been accessible to Ca^2+^ imaging. In the future, we expect that such Na^+^ imaging data will allow us to further constrain simulations of dendritic integration in GCs or of post-synaptic GC spine signals (as in Bywalez et al., 2015), and yield insights into the role of Na^+^ currents in reciprocal processing at the dendrodendritic MC-GC or TC-GC synapse.

## Author Contributions

All authors take responsibility for the integrity of the data and the accuracy of the data analysis. Study concept and design: CR and VE. Calibration: NG and TO-J. Acquisition of other data: TO-J. Simulations: SS. Analysis and interpretation of data: all. Figure preparation: TO-J, NG, and VE. Drafting of manuscript: VE. Editing of manuscript: all authors. Obtained funding: VE and CR.

## Conflict of Interest Statement

The authors declare that the research was conducted in the absence of any commercial or financial relationships that could be construed as a potential conflict of interest.

## Footnotes

1 https://senselab.med.yale.edu/ModelDB

2 http://vassarstats.net/

## Abbreviations

EPL: external plexiform layer
GC: granule cell
MC: mitral cell
MCL: mitral cell layer
SVR: surface to volume ratio
TC: tufted cell

## References

[B1] Bender K. J. Trussell L. O. (2009). Axon initial segment Ca^2+^ channels influence action potential generation and timing. *Neuron* 61 259–271. 10.1016/j.neuron.2008.12.004 19186168 PMC2730113

[B2] Burton S. D. Urban N. N. (2015). Rapid feedforward inhibition and asynchronous excitation regulate granule cell activity in the mammalian main olfactory bulb. *J. Neurosci.* 35 14103–14122. 10.1523/JNEUROSCI.0746-15.2015 26490853 PMC4683680

[B3] Bywalez W. G. Patirniche D. Rupprecht V. Stemmler M. Herz A. V. Palfi D. (2015). Local postsynaptic voltage-gated sodium channel activation in dendritic spines of olfactory bulb granule cells. *Neuron* 85 590–601. 10.1016/j.neuron.2014.12.051 25619656

[B4] Carnevale T. Hines M. L. (2006). *The NEURON Book.* Cambridge: Cambridge University Press.

[B5] Chen W. R. Midtgaard J. Shepherd G. M. (1997). Forward and backward propagation of dendritic impulses and their synaptic control in mitral cells. *Science* 278 463–467. 10.1126/science.278.5337.463 9334305

[B6] Christie J. M. Westbrook G. L. (2003). Regulation of backpropagating action potentials in mitral cell lateral dendrites by A-type potassium currents. *J. Neurophysiol.* 89 2466–2472. 10.1152/jn.00997.2002 12740404

[B7] Debarbieux F. Audinat E. Charpak S. (2003). Action potential propagation in dendrites of rat mitral cells in vivo. *J. Neurosci.* 23 5553–5560. 12843256 10.1523/JNEUROSCI.23-13-05553.2003 PMC6741248

[B8] Djurisic M. Antic S. Chen W. R. Zecevic D. (2004). Voltage imaging from dendrites of mitral cells: EPSP attenuation and spike trigger zones. *J. Neurosci.* 24 6703–6714. 10.1523/jneurosci.0307-04.2004 15282273 PMC6729725

[B9] Egger V. (2008). Synaptic sodium spikes trigger long-lasting depolarizations and slow calcium entry in rat olfactory bulb granule cells. *Eur. J. Neurosci.* 27 2066–2075. 10.1111/j.1460-9568.2008.06170.x 18412627

[B10] Egger V. Stroh O. (2009). Calcium buffering in rodent olfactory bulb granule cells and mitral cells. *J. Physiol.* 587(Pt 18) 4467–4479. 10.1113/jphysiol.2009.174540 19635818 PMC2766651

[B11] Egger V. Svoboda K. Mainen Z. F. (2003). Mechanisms of lateral inhibition in the olfactory bulb: efficiency and modulation of spike-evoked calcium influx into granule cells. *J. Neurosci.* 23 7551–7558. 12930793 10.1523/JNEUROSCI.23-20-07551.2003 PMC6740749

[B12] Fleidervish I. A. Lasser-Ross N. Gutnick M. J. Ross W. N. (2010). Na^+^ imaging reveals little difference in action potential-evoked Na^+^ influx between axon and soma. *Nat. Neurosci.* 13 852–860. 10.1038/nn.2574 20543843 PMC3102307

[B13] Friedrich R. W. Wiechert M. T. (2014). Neuronal circuits and computations: pattern decorrelation in the olfactory bulb. *FEBS Lett.* 588 2504–2513. 10.1016/j.febslet.2014.05.055 24911205

[B14] Fukunaga I. Berning M. Kollo M. Schmaltz A. Schaefer A. T. (2012). Two distinct channels of olfactory bulb output. *Neuron* 75 320–329. 10.1016/j.neuron.2012.05.017 22841316

[B15] Hines M. L. Davison A. P. Muller E. (2009). NEURON and Python. *Front. Neuroinform.* 3:1 10.3389/neuro.11.001.2009 PMC2636686 19198661

[B16] Hu W. Tian C. Li T. Yang M. Hou H. Shu Y. (2009). Distinct contributions of Na(v)1.6 and Na(v)1.2 in action potential initiation and backpropagation. *Nat. Neurosci.* 12 996–1002. 10.1038/nn.2359 19633666

[B17] Igarashi K. M. Ieki N. An M. Yamaguchi Y. Nagayama S. Kobayakawa K. (2012). Parallel mitral and tufted cell pathways route distinct odor information to different targets in the olfactory cortex. *J. Neurosci.* 32 7970–7985. 10.1523/JNEUROSCI.0154-12.2012 22674272 PMC3636718

[B18] Jaffe D. B. Johnston D. Lasser-Ross N. Lisman J. E. Miyakawa H. Ross W. N. (1992). The spread of Na^+^ spikes determines the pattern of dendritic Ca^2+^ entry into hippocampal neurons. *Nature* 357 244–246. 10.1038/357244a0 1350327

[B19] Karus C. Mondragao M. A. Ziemens D. Rose C. R. (2015). Astrocytes restrict discharge duration and neuronal sodium loads during recurrent network activity. *Glia* 63 936–957. 10.1002/glia.22793 25639699

[B20] Kim S. Guzman S. J. Hu H. Jonas P. (2012). Active dendrites support efficient initiation of dendritic spikes in hippocampal CA3 pyramidal neurons. *Nat. Neurosci.* 15 600–606. 10.1038/nn.3060 22388958 PMC3617474

[B21] Kole M. H. P. Ilschner S. U. Kampa B. M. Williams S. R. Ruben P. C. Stuart G. J. (2008). Action potential generation requires a high sodium channel density in the axon initial segment. *Nat. Neurosci.* 11 178–186. 10.1038/nn2040 18204443

[B22] Labarrera C. London M. Angelo K. (2013). Tonic inhibition sets the state of excitability in olfactory bulb granule cells. *J. Physiol.* 591(Pt 7) 1841–1850. 10.1113/jphysiol.2012.241851 23318869 PMC3624854

[B23] Langer J. Rose C. R. (2009). Synaptically induced sodium signals in hippocampal astrocytes in situ. *J. Physiol.* 587(Pt 24) 5859–5877. 10.1113/jphysiol.2009.182279 19858225 PMC2808545

[B24] Longair M. H. Baker D. A. Armstrong J. D. (2011). Simple Neurite Tracer: open source software for reconstruction, visualization and analysis of neuronal processes. *Bioinformatics* 27 2453–2454. 10.1093/bioinformatics/btr390 21727141

[B25] Lorincz A. Nusser Z. (2010). Molecular identity of dendritic voltage-gated sodium channels. *Science* 328 906–909. 10.1126/science.1187958 20466935 PMC3546315

[B26] Luo M. Katz L. C. (2001). Response correlation maps of neurons in the mammalian olfactory bulb. *Neuron* 32 1165–1179. 10.1016/S0896-6273(01)00537-2 11754845

[B27] Magee J. (2008). ”Dendritic voltage-gated channels”, in *Dendrites* eds Stuart G. Spruston N. Häusser M. (Oxford Oxford University Press) 225-250.

[B28] Manabe H. Mori K. (2013). Sniff rhythm-paced fast and slow gamma-oscillations in the olfactory bulb: relation to tufted and mitral cells and behavioral states. *J. Neurophysiol.* 110 1593–1599. 10.1152/jn.00379.2013 23864376

[B29] Maravall M. Mainen Z. F. Sabatini B. L. Svoboda K. (2000). Estimating intracellular calcium concentrations and buffering without wavelength ratioing. *Biophys. J.* 78 2655–2667. 10.1016/S0006-3495(00)76809-3 10777761 PMC1300854

[B30] Margrie T. W. Sakmann B. Urban N. N. (2001). Action potential propagation in mitral cell lateral dendrites is decremental and controls recurrent and lateral inhibition in the mammalian olfactory bulb. *Proc. Natl. Acad. Sci. U.S.A.* 98 319–324. 10.1073/pnas.011523098 11120888 PMC14588

[B31] Martina M. Vida I. Jonas P. (2000). Distal initiation and active propagation of action potentials in interneuron dendrites. *Science* 287 295–300. 10.1126/science.287.5451.295 10634782

[B32] Meier S. D. Kovalchuk Y. Rose C. R. (2006). Properties of the new fluorescent Na^+^ indicator CoroNa Green: comparison with SBFI and confocal Na^+^ imaging. *J. Neurosci. Methods* 155 251–259. 10.1016/j.jneumeth.2006.01.009 16488020

[B33] Merkle F. T. Fuentealba L. C. Sanders T. A. Magno L. Kessaris N. Alvarez-Buylla A. (2014). Adult neural stem cells in distinct microdomains generate previously unknown interneuron types. *Nat. Neurosci.* 17 207–214. 10.1038/nn.3610 24362763 PMC4100623

[B34] Metsalu T. Vilo J. (2015). ClustVis: a web tool for visualizing clustering of multivariate data using Principal Component Analysis and heatmap. *Nucleic Acids Res.* 43 W566–W570. 10.1093/nar/gkv468 25969447 PMC4489295

[B35] Miyazaki K. Ross W. N. (2015). Simultaneous sodium and calcium imaging from dendrites and axons. *eNeuro* 2:ENEURO.0092-15.2015 10.1523/ENEURO.0092-15.2015 PMC4699831 26730401

[B36] Mondragao M. A. Schmidt H. Kleinhans C. Langer J. Kafitz K. W. Rose C. R. (2016). Extrusion versus diffusion: mechanisms for recovery from sodium loads in mouse CA1 pyramidal neurons. *J. Physiol.* 594 5507–5527. 10.1113/jp272431 27080107 PMC5043027

[B37] Mori K. Kishi K. Ojima H. (1983). Distribution of dendrites of mitral, displaced mitral, tufted, and granule cells in the rabbit olfactory bulb. *J. Comp. Neurol.* 219 339–355. 10.1002/cne.902190308 6619342

[B38] Mori K. Takagi S. F. (1978). An intracellular study of dendrodendritic inhibitory synapses on mitral cells in the rabbit olfactory bulb. *J. Physiol.* 279 569–588. 10.1113/jphysiol.1978.sp012362 671363 PMC1282633

[B39] Nagayama S. Homma R. Imamura F. (2014). Neuronal organization of olfactory bulb circuits. *Front. Neural Circuits* 8:98 10.3389/fncir.2014.00098 PMC4153298 25232305

[B40] Neher E. Augustine G. J. (1992). Calcium gradients and buffers in bovine chromaffin cells. *J. Physiol.* 450 273–301. 10.1113/jphysiol.1992.sp019127 1331424 PMC1176122

[B41] Orona E. Scott J. W. Rainer E. C. (1983). Different granule cell populations innervate superficial and deep regions of the external plexiform layer in rat olfactory bulb. *J. Comp. Neurol.* 217 227–237. 10.1002/cne.902170209 6886054

[B42] Pinato G. Midtgaard J. (2005). Dendritic sodium spikelets and low-threshold calcium spikes in turtle olfactory bulb granule cells. *J. Neurophysiol.* 93 1285–1294. 10.1152/jn.00807.2004 15483062

[B43] Price J. L. Powell T. P. (1970). The mitral and short axon cells of the olfactory bulb. *J. Cell Sci.* 7 631–651. 5492279 10.1242/jcs.7.3.631

[B44] Rose C. R. Konnerth A. (2001). NMDA receptor-mediated Na^+^ signals in spines and dendrites. *J. Neurosci.* 21 4207–4214. 11404406 10.1523/JNEUROSCI.21-12-04207.2001 PMC6762772

[B45] Rose C. R. Kovalchuk Y. Eilers J. Konnerth A. (1999). Two-photon Na^+^ imaging in spines and fine dendrites of central neurons. *Pflugers Arch.* 439 201–207. 10.1007/s004249900123 10651018

[B46] Rose C. R. Ransom B. R. (1996). Intracellular sodium homeostasis in rat hippocampal astrocytes. *J. Physiol.* 491(Pt 2) 291–305. 8866855 10.1113/jphysiol.1996.sp021216 PMC1158726

[B47] Ross W. N. Miyakawa H. Lev-Ram V. Lasser-Ross N. Lisman J. Jaffe D. (1993). Dendritic excitability in CNS neurons: insights from dynamic calcium and sodium imaging in single cells. *Jpn. J. Physiol.* 43(Suppl. 1) S83–S89. 8271520

[B48] Sabatini B. L. Maravall M. Svoboda K. (2001). Ca^2+^ signaling in dendritic spines. *Curr. Opin. Neurobiol.* 11 349–356. 10.1016/s0959-4388(00)00218-x 11399434

[B49] Schreiner A. Rose C. (2012). “Quantitative imaging in intracellular sodium,” in *Current Microscopy Contributions to Advances in Science and Technology* ed. Méndez-Vilas A. (Badajoz: Formatex Research Center) 119–129.

[B50] Scott R. S. Henneberger C. Padmashri R. Anders S. Jensen T. P. Rusakov D. A. (2014). Neuronal adaptation involves rapid expansion of the action potential initiation site. *Nat. Commun.* 5 3817 10.1038/ncomms4817 PMC4050282 24851940

[B51] Sheperd G. M. Greer C. A. Haberly L. B. (1990). *The Synaptic Organization of the Brain.* New York, NY: Oxford University Press.

[B52] Wachowiak M. Shipley M. T. (2006). Coding and synaptic processing of sensory information in the glomerular layer of the olfactory bulb. *Semin. Cell Dev. Biol.* 17 411–423. 10.1016/j.semcdb.2006.04.007 16765614

[B53] Wellis D. P. Scott J. W. (1990). Intracellular responses of identified rat olfactory bulb interneurons to electrical and odor stimulation. *J. Neurophysiol.* 64 932–947. 2230935 10.1152/jn.1990.64.3.932

[B54] Woolf T. B. Shepherd G. M. Greer C. A. (1991). Serial reconstructions of granule cell spines in the mammalian olfactory bulb. *Synapse* 7 181–192. 10.1002/syn.890070303 1882328

[B55] Xiong W. Chen W. R. (2002). Dynamic gating of spike propagation in the mitral cell lateral dendrites. *Neuron* 34 115–126. 10.1016/S0896-6273(02)00628-1 11931746

[B56] Yuste R. Majewska A. Holthoff K. (2000). From form to function: calcium compartmentalization in dendritic spines. *Nat. Neurosci.* 3 653–659. 10.1038/76609 10862697

[B57] Zelles T. Boyd J. D. Hardy A. B. Delaney K. R. (2006). Branch-specific Ca^2+^ influx from Na^+^-dependent dendritic spikes in olfactory granule cells. *J. Neurosci.* 26 30–40. 10.1523/JNEUROSCI.1419-05.2006 16399670 PMC6674300

[B58] Zylbertal A. Kahan A. Ben-Shaul Y. Yarom Y. Wagner S. (2015). Prolonged intracellular Na^+^ dynamics govern electrical activity in accessory olfactory bulb mitral cells. *PLoS Biol.* 13:e1002319 10.1371/journal.pbio.1002319 PMC4684409 26674618

